# Reducing the use of high dose antipsychotic medication in acute adult inpatient psychiatric units

**DOI:** 10.1192/bjo.2021.893

**Published:** 2021-06-18

**Authors:** Shay-Anne Pantall, Sarah Warwicker, Lisa Brownell

**Affiliations:** 1 Birmingham and Solihull Mental Health NHS Foundation Trust; 2 University of Birmingham

## Abstract

**Aims:**

To evaluate the use of antipsychotics, and high dose antipsychotic treatment (HDAT) in psychiatric inpatient units

**Background:**

The Royal College of Psychiatrists published a consensus statement on high dose antipsychotic medication in October 1993. Such treatment carries an increased risk of adverse effects including towards ventricular tachycardia and sudden death.

**Method:**

A retrospective case note review of all male patients on acute adult inpatient units in a psychiatric hospital in South Birmingham on a date in June 2018 (n = 45) including review of electronic patient records and prescriptions. This was compared with the results of an earlier study, with identical methods, undertaken in June 2015.

**Result:**

- In both 2015 and 2018, only a minority of patients (20% and 11% respectively) were informal.
- In both 2015 and 2018, the majority of inpatients had a diagnosis of schizophrenia (54% and 67%)
- In both 2015 and 2018, 93% inpatients were prescribed antipsychotic medication.
- In 2015, 56% patients were prescribed HDAT. This reduced in 2018 to 16%.
- This reduction in use of HDAT was almost entirely due to a reduction in the prescription of PRN antipsychotic medication.
- In terms of regularly prescribed antipsychotic medication, in both years, the most commonly prescribed drug was flupentixol, with a range of other second generation oral and long acting medications being prescribed, usually at doses within BNF limits.

Between the two years, there was a substantial change in the prescribing of PRN antipsychotics. In 2015, 59% individuals were prescribed at least one PRN antipsychotic (27% were prescribed two). In 2018, this reduced to 40% prescribed at least one, and only 2% being prescribed 2 PRN antipsychotics. In both years, oral quetiapine was a common choice (39% patients in 2015 prescribed oral quetiapine, and 34% in 2018). In 2015, 39% patients were prescribed oral or intramuscular aripiprazole, while this reduced to 7% in 2018.

**Conclusion:**

The vast majority of psychiatric inpatients were being prescribed antipsychotic medication. Prescription of high dose antipsychotic medication was common in 2015, and this was largely attributable to high levels of prescribing of PRN antipsychotics. Following an educational programme for junior doctors and ward nurses, and the introduction of electronic prescribing, we achieved a significant change in practice, particularly in the prescribing of PRN antipsychotics, which has reduced our patients’ risk of receiving high dose antipsychotic medication.

